# Risk stratification in fecal immunochemical test-based colorectal cancer screening: Public acceptance and experiences with tailored invitation intervals

**DOI:** 10.1016/j.pmedr.2025.103310

**Published:** 2025-11-12

**Authors:** Brenda J. van Stigt, Elyse E.C. Rijnders, Lucie de Jonge, Iris Lansdorp-Vogelaar, Esther Toes-Zoutendijk, on behalf of the Dutch colorectal cancer screening working group, Hilliene J. van de Schootbrugge-Vandermeer, Manon C.W. Spaander, Anneke J. van Vuuren, Evelien Dekker, Folkert J. van Kemenade, Iris D. Nagtegaal, Monique E. van Leerdam

**Affiliations:** a Department of Public Health, Erasmus MC University Medical Centre, Rotterdam, The Netherlands; b Department of Gastroenterology and Hepatology, Erasmus MC University Medical Centre, Rotterdam, The Netherlands; c Department of Gastroenterology and Hepatology, Amsterdam University Medical Centre, Amsterdam, The Netherlands; d Department of Pathology, Erasmus MC University Medical Centre, Rotterdam, The Netherlands; e Department of Pathology, Radboud University Medical Centre, Nijmegen, The Netherlands; f Department of Gastrointestinal Oncology, Antoni van Leeuwenhoek Hospital, Amsterdam, The Netherlands; g Department of Gastroenterology and Hepatology, Leiden University Medical Centre, Leiden, The Netherlands; a Department of Public Health, Erasmus MC University Medical Centre; Rotterdam, the Netherlands; b Department of Gastroenterology and Hepatology, Erasmus MC University Medical Centre, Rotterdam, the Netherlands

**Keywords:** Colorectal cancer, Colorectal cancer screening, Personalized cancer screening, Public acceptance, Risk stratification

## Abstract

**Objective:**

Risk stratification based on prior fecal Hemoglobin (f-Hb) concentrations offers potential to enhance the effectiveness of fecal immunochemical test (FIT)-based colorectal cancer (CRC) screening programs. Acceptance of tailored invitation intervals has not been assessed in individuals undergoing risk-stratified CRC screening.

**Methods:**

We conducted five semi-structured focus groups with participants from the Dutch risk-stratified CRC screening trial (PERFECT-FIT), to explore their experiences with tailored invitation intervals. Study participants included thirteen individuals assigned an extended (three-year) invitation interval and eleven individuals assigned a shorter (one-year) invitation interval based on their prior f-Hb concentration. Additionally, four individual interviews were conducted with individuals who had dropped out of the trial after being assigned a three-year interval. All data were collected between January and June 2023. Transcripts were thematically analyzed using ATLAS.ti.

**Results:**

Intensified screening was preferred over less intensive screening, driven by the perceived low burden of FIT-based screening and a desire to detect lesions as early as possible. Shorter intervals were therefore readily accepted. Extended intervals raised concerns about missed lesions but were largely accepted as individuals felt reassured by their low-risk classification. Individuals were less concerned about risk-stratified intervals when implemented nationwide, trusting the provider would only implement these after scientific evaluation of potential risks.

**Conclusions:**

Our findings support the acceptance of tailored invitation intervals within a population-based CRC screening program. This acceptance appears to be influenced by participants' high trust in the screening provider, emphasizing the importance of maintaining this trust. Clear communication of a changing screening approach, especially the reporting of negative FIT-results, may facilitate the transition from uniform to risk-stratified screening.

## Highlights

• Shorter intervals were readily accepted as individuals preferred more screening.
• Low-risk status and trust in provider supported acceptance of extended intervals.
• Screening wasn't perceived as burdensome, limiting understanding of extended intervals.
• Risk stratification seems best understood in the light of equal resource distribution.
• Changes to the reporting of stool test results should be clearly explained.

## 1 Introduction

Colorectal cancer (CRC) screening programs have been successfully implemented worldwide (Schreuders et al., 2015; Breekveldt et al., 2022; Chiu et al., 2015; Ventura et al., 2014). Risk stratification offers potential to enhance the effectiveness of screening programs by optimizing the harm-to-benefit ratio of screening. For fecal immunochemical test (FIT)-based programs specifically, stratifying screening strategies based on prior fecal hemoglobin (f-Hb) concentrations appears to be a promising approach (Lansdorp-Vogelaar et al., 2022). While FIT results are often dichotomously classified as positive or negative based on a predefined cut-off, quantitative f-Hb concentrations hold valuable additional information. Prior research indicates that quantitative f-Hb concentrations are positively associated with the future detection of advanced adenomas and CRC, also among those with a negative FIT (Digby et al., 2017; Kooyker et al., 2020; Senore et al., 2020; Buron et al., 2019; van den Berg et al., 2025). Individuals with f-Hb concentrations just below the FIT cut-off may therefore benefit from a shorter invitation interval, increasing the likelihood of early detection and successful treatment. Conversely, individuals without detectable f-Hb may benefit from an extended invitation interval, minimizing unnecessary interventions and associated harms.

Changes to a screening program may affect individuals' perception and adherence to screening recommendations. Evaluating public receptiveness is therefore crucial before implementing risk-stratified CRC screening in nationwide programs. Prior research already showed that extended invitation intervals in cancer screening may be less acceptable than shorter intervals (Dunlop et al., 2021; Kvernrød et al., 2024; Rainey et al., 2020; Kelley Jones et al., 2024; Loft et al., 2024; Ghanouni et al., 2020; Taylor et al., 2024). However, these studies have been conducted in hypothetical scenarios, which may not accurately reflect real-world experiences. This study aims to evaluate individuals' experiences and perceptions of being assigned tailored invitation intervals in an ongoing risk-stratified FIT-based CRC screening program.

## 2 Methods

### 2.1 Study design

**Fig. 1:**
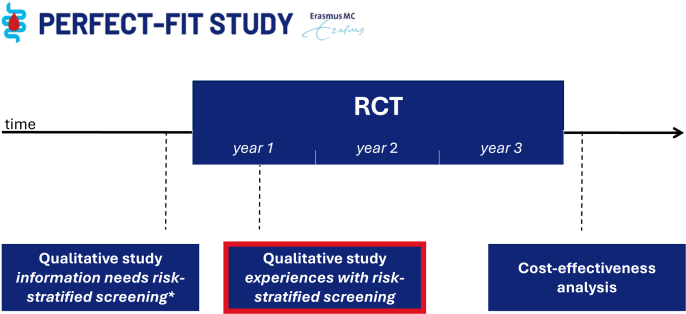
Timeline of the PERFECT-FIT study, highlighting the present qualitative study. Abbreviation: RCT = Randomized Controlled Trial. * Results published (Toes-Zoutendijk et al., 2023)

The mixed-method PERFECT-FIT study evaluates the feasibility, acceptance and cost-effectiveness of risk-stratified CRC screening. This study is described in more detail elsewhere (Breekveldt et al., 2023). In short, it comprises a nationwide randomized controlled trial (RCT) within the Dutch CRC screening program, complemented by qualitative studies and a cost-effectiveness analysis (Fig. 1). The RCT started in 2023 and is currently ongoing. Within the intervention arm of this RCT, individuals with a negative FIT (<47 μg Hb/g feces) are assigned an invitation interval based on the f-Hb concentration measured in their last FIT. Invitation intervals refer to the period between a negative FIT result and the invitation for the next FIT-screening within the program. Intervals of three, two and one year(s) are allocated to individuals with f-Hb levels of 0 μg, >0–15 μg and > 15–47 μg Hb/g feces, respectively.

This qualitative study is part of the PERFECT-FIT study, and includes both focus groups and individual interviews with RCT participants. It's primary aim was to explore individuals' experiences, perceptions, and opinions regarding tailored invitation intervals. Focus groups were conducted as this method helps to gain insight into individuals' thoughts and experiences by allowing them to build on each other's responses (Denny and Weckesser, 2019; Kitzinger, 1995; Skovdal and Cornish, 2015). Some individuals withdrew from participation in the RCT after they were assigned a three-year interval. As thoughts surrounding the decision to withdraw could provide valuable insights into the acceptance of risk-stratified screening, additional interviews were conducted among these individuals. Individual interviews were preferred over focus groups for this subgroup, as withdrawing could be perceived as a sensitive topic, and we wanted to ensure individuals felt comfortable speaking freely. Individuals received a €25 gift voucher after participation in either a focus group or individual interview.

### 2.2 Recruitment

The study population consisted of participants from the intervention arm of the PERFECT-FIT RCT. Inclusion criteria for the RCT required participants to have received a negative FIT result (<47 μg Hb/g feces) in the previous round of the Dutch CRC screening program (Breekveldt et al., 2023). To ensure eligibility for at least one additional screening round following participation in the RCT, only individuals aged ≤72 years were included. Additional inclusion criteria for our qualitative study included the Dutch language proficiency and access to a mobile device or computer. The RCT invitation letter included information on the RCT's background and aim, as well as the advantages and disadvantages of participation (Supplementary file A). An informed consent form for the qualitative study was added to the invitation, allowing individuals to give permission to be contacted for the qualitative study when consenting to participate in the RCT (Supplementary file B).

After random allocation to the intervention or control arm, individuals in the intervention arm assigned a one- or three-year interval who had consented to qualitative research were randomly selected to participate in focus groups. Individuals who had consented to the qualitative study but withdrew from the RCT after interval allocation, were approached to participate in individual interviews. We aimed to include four to five individuals per focus group session, aiming for diversity in age and sex. Recruitment continued until data saturation was reached, meaning no new themes emerged from additional focus groups or interviews (Krueger, 2014).

### 2.3 Data collection

Focus groups and individual interviews were conducted between January and June 2023 and lasted approximately 90 min (focus groups) or 30 min (individual interviews). All sessions were conducted online, via Teams (Microsoft Corporation, Washington), and were audio recorded with permission of the participants. Both focus groups and individual interviews were semi-structured, guided by interview guides to provide structure and consistency across sessions (Supplementary file C and D). The interview guides were collaboratively developed by the study team, which included experts in public health, cancer screening, and qualitative research, and were informed by our study objectives as well as insights from previous research on risk-stratified screening within a similar population (Toes-Zoutendijk et al., 2023). All focus group sessions were moderated by two female epidemiologists (LdJ, MSc; ETZ, PhD) from the Erasmus MC University Medical Centre. Both had experience conducting focus groups on risk-stratified CRC screening in a similar population. Individual interviews were conducted by LdJ and either ETZ or HvdSV (female epidemiologist, MSc)). A third researcher was present in all sessions to record conversations.

### 2.4 Data analysis

Data were anonymized, transcribed non-verbatim and analyzed following Braun and Clarke's six-step thematic analysis approach (Braun and Clarke, 2006). Data were independently analyzed and inductively coded by two researchers (BvS and ER), who discussed codes before agreeing on final themes and subthemes. Themes, subthemes and corresponding quotes were translated to English. Reporting followed the consolidated criteria for reporting qualitative research (COREQ) (Tong et al., 2007).

Data were analyzed using the qualitative research software package ATLAS.ti 24 (Scientific Software Development GmbH, Berlin).

### 2.5 Ethical considerations

The PERFECT-FIT study was approved by the Dutch Ministry of Health, Welfare, and Sport, following the recommendation of the Dutch Health Council. A Population Screening Act permit was issued, confirming that the study was scientifically sound, complied with legal standards for medical treatment, and had a favorable risk-benefit profile. Additional data collection not covered by the initial study protocol (i.e. conducting individual interviews with individuals who had withdrawn from the RCT) was approved by the Dutch Health Council. The study adhered to the principles of the Declaration of Helsinki (World Medical Association, 2013).

## 3 Results

### 3.1 Study population

The population consisted of 28 individuals with a median age of 64 (interquartile range: 60–69), of which a slight majority was female (57.1 %) (Table 1). Of all individuals, 13 were assigned a one-year interval (46.4 %) and 15 were assigned a three-year interval (54.6 %). A total of five focus groups and four individual interviews were conducted. A comprehensive flowchart of the study population is shown in Supplementary Fig. 1.

**Table 1:** Characteristics of the study population (*n* = 28), comprising of individuals assigned a risk-stratified invitation interval within the Dutch colorectal cancer screening program (2023).

	**Participants one-year interval (*n* = 13)**	**Participants three-year interval (*n* = 11)**	**Dropouts three-year interval (*n* = 4)**	**Total (*n* = 28)**
**Male (*n*, %)**	6 (46.2)	4 (36.4)	2 (50.0)	12 (42.9)
**Age (median, IQR)**	64 (59–67)	68 (61–70)	60 (60–64)	64 (60–69)

Abbreviation: IQR = Interquartile Range.

### 3.2 Themes and subthemes

**Fig. 2:**
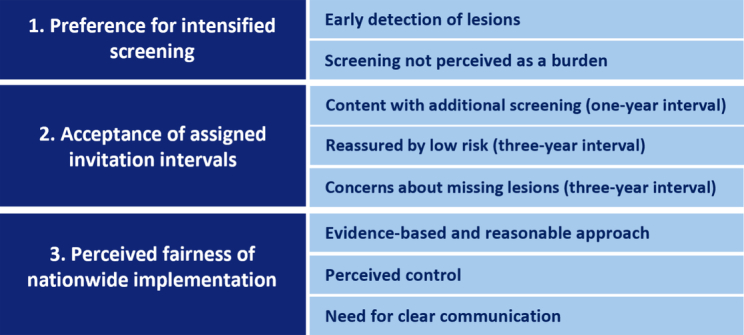
Overview of themes and subthemes identified from focus groups and individual interviews with individuals assigned a risk-stratified invitation intervals in the Dutch colorectal cancer screening program (2023).

Three themes and eight subthemes emerged from our data (Fig. 2). Main themes included 1) Preference for intensified screening, 2) Acceptance of assigned invitation intervals, and 3) Perceived fairness of nationwide implementation.

### 3.3 Theme 1: Preference for intensified screening.

#### 3.3.1 Early detection of lesions

**Fig. 3:**
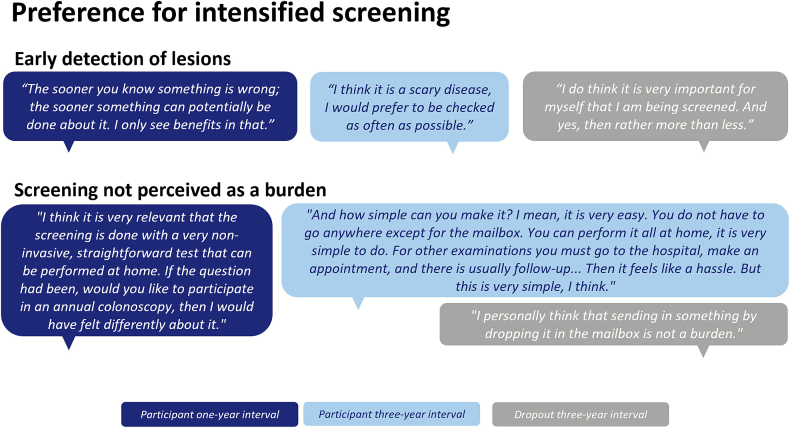

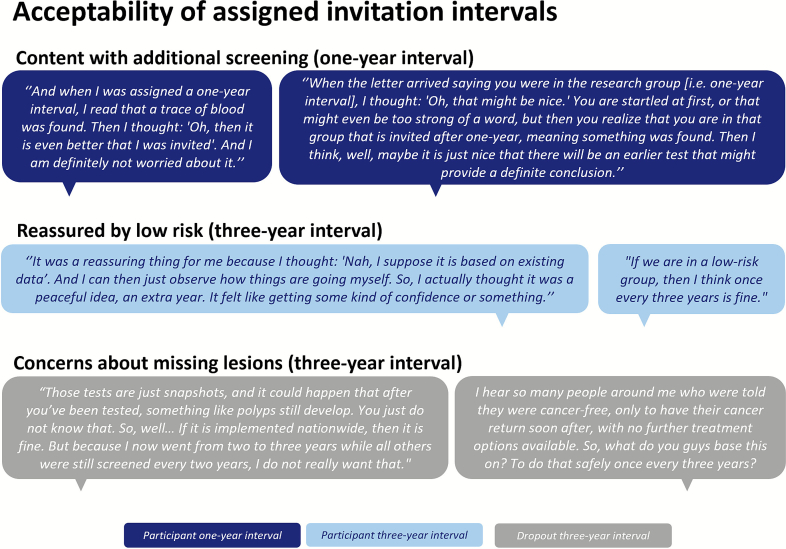

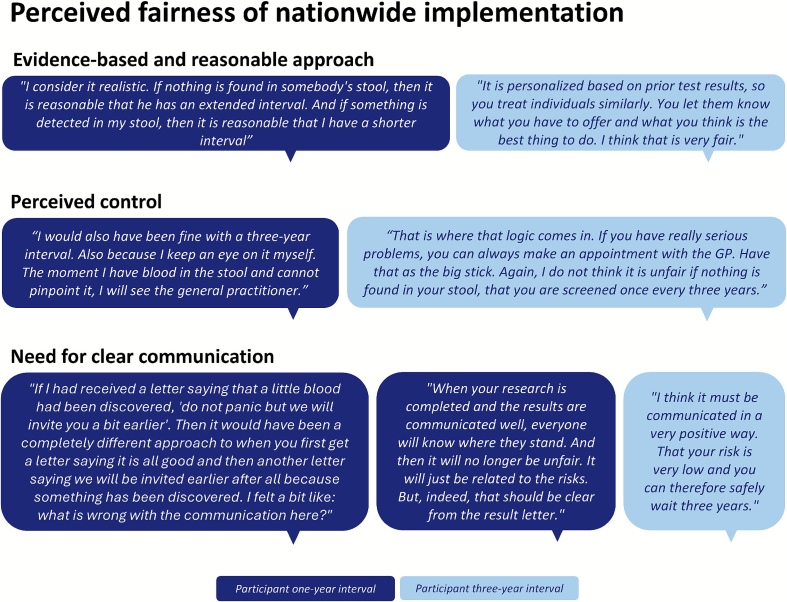
**a.** Quotations on theme 1 ‘Preference for intensified screening’ and corresponding subthemes identified from focus groups and individual interviews with individuals assigned to risk-stratified invitation intervals in the Dutch colorectal cancer screening program (2023). **b:** Quotations on theme 2 ‘Acceptability of assigned invitation intervals and corresponding subthemes identified from focus groups and individual interviews with individuals assigned to risk-stratified invitation intervals in the Dutch colorectal cancer screening program (2023). **c**: Quotations on theme 3 ‘Perceived fairness of nationwide implementation’ and corresponding subthemes identified from focus groups and individual interviews with individuals assigned to risk-stratified invitation intervals in the Dutch colorectal cancer screening program (2023).

Overall, individuals consistently expressed a clear preference for more intensive over less intensive screening, regardless of their assigned interval. This preference was primarily driven by the desire to detect and treat potential lesions as early as possible (Fig. 3a). Some individuals were particularly cautious due to personal or familial cancer, which increased their awareness of the importance of regular screening. Others would like to be monitored more closely due to experiencing gut-related symptoms. The preference for more intensive screening was also reflected in suggestions provided to improve the current screening program, such as to start screening at a younger age, to continue screening beyond age 75 and to offer additional screening for individuals with a history of gut-related symptoms. Moreover, some individuals indicated that their decision to participate in the RCT was motivated by the opportunity to undergo more frequent screening.

#### 3.3.2 Screening not perceived as a burden

Individuals' preference to be screened more often was facilitated by their positive experiences with the Dutch CRC screening program. Individuals did not perceive participating in the program as burdensome. A key factor in this was the use of the FIT as the initial screening test, particularly because it is a relatively simple test that can be performed at home (Fig. 3a). Accordingly, it was discussed that annual screening would be less accepted if it involved an invasive procedure, such as a colonoscopy.

### 3.4 Theme 2: Acceptance of assigned invitation intervals

#### 3.4.1 Content with additional screening (one-year interval)

Some individuals assigned a one-year interval reported initially feeling startled when they were informed some blood was detected in their stool, placing them at higher risk. They had previously assumed that a negative FIT result meant no blood at all had been detected. The most prominent emotion, however, was that individuals were glad they would be screened more intensively, especially considering their higher risk (Fig. 3b). Individuals felt mainly reassured knowing they would be monitored more closely, allowing potential abnormalities to be detected in time. This additional screening provided a sense of security. Moreover, some individuals noted that awareness of their CRC risk increased their motivation to adhere to screening.

#### 3.4.2 Reassured by low risk (three-year interval)

A perspective widely shared among individuals assigned a three-year interval was the feeling of reassurance from knowing that no blood was detected in their stool, indicating a low CRC risk (Fig. 3b). This knowledge enhanced their confidence in their health, making them accept an extended invitation interval. Although some individuals indicated they initially were unsure about an extended interval, they largely trusted the research team would not let them face irresponsible risks, and realized it was a positive indication they were healthy enough to be screened less intensively.

#### 3.4.3 Concerns about missing lesions (three-year interval)

Not all individuals felt comfortable with an extended interval. Some found it difficult to accept they would be screened less intensively than others, particularly due to concerns that extended intervals might lead to missed cancers (Fig. 3b). This concern was especially prevalent among those who withdrew from the RCT after being assigned a three-year interval. Most thought they would be screened more intensively when participating in the RCT.

Concerns about three-year intervals were largely confined to the research setting. Several participants who withdrew from participation in the trial stated they would accept extended intervals if implemented in the national screening program. This acceptance was primarily driven by trust in the provider, believing that potential risks of risk-stratified screening would be thoroughly evaluated before nationwide implementation.

### 3.5 Theme 3: Perceived fairness of nationwide implementation

#### 3.5.1 Evidence-based and reasonable approach

Individuals generally understood the concept of risk-stratified screening. It was considered perfectly reasonable to offer more intensive screening to those at higher risk, while reducing screening intensity for those at lower risk (Fig. 3c). Nearly all individuals viewed the nationwide implementation of tailored intervals as fair. An important condition, however, was that the stratification method was scientifically proven. Accordingly, some expressed doubts about the fairness of risk-based screening within a research trial, where the method's effectiveness is still being evaluated. Nationwide implementation was also considered fairer than a trial as it ensures equal treatment for individuals with similar FIT results. However, few individuals were uncertain about the fairness of nationwide risk-stratified screening, feeling they lacked the expertise to judge such decisions.

#### 3.5.2 Perceived control

The concept of self-determination was discussed several times in relation to the fairness of risk-stratified intervals. Some individuals felt that the ability to monitor gut-related symptoms and changes in bowel habits between screening tests provide a sense of security and control (Fig. 3c). For them, the fact that individuals can always visit their general practitioner if they notice blood in their stool or feel something is off facilitated the acceptance and fairness of extended intervals. However, some did not share this perspective, acknowledging that polyps or cancer can develop without noticeable symptoms or visible blood loss.

#### 3.5.3 Need for clear communication

While some individuals mentioned they might not even notice it when their invitation interval would change, individuals agreed that any changes should be clearly communicated to the public. This was also identified as a condition for the perceived fairness of risk-stratified screening (Fig. 3c). Several suggestions were made to improve information material. Firstly, many found the information too lengthy and suggested summarizing key points at the top of the letter to make the main message more accessible.

Secondly, individuals emphasized the need for consistent and transparent communication about the meaning of negative FIT results. Before the RCT, most believed a negative FIT meant that no blood at all was detected in their stool. They were therefore surprised to learn this was not always the case. Those assigned a one-year interval were particularly confused upon learning that traces of blood had been found, as this seemed to contradict the message they received from the screening organization following their negative FIT. This information stated that no follow-up examination was required, while the study information mentioned traces of blood in the stool which led to a shorter screening interval. This inconsistency was seen as confusing and undesirable, with some individuals feeling that information had been withheld from them.

Thirdly, individuals suggested highlighting the benefits of risk-stratified screening, particularly when assigning extended intervals. They felt that emphasizing the low risk of those given a three-year interval, and explaining that this makes extended intervals safe, could support the acceptance of nationwide risk-stratified screening.

Lastly, individuals expressed a need for more information on potential warning signs, such as changes in bowel habits or gastrointestinal complaints. They suggested including a list in the information materials to help individuals know when to seek medical advice between screening tests.

## 4 Discussion

Our findings demonstrate high acceptance of tailored invitation intervals among individuals in a risk-stratified CRC screening trial in the Netherlands. Individuals expressed a strong preference for intensified screening due to the perceived low burden of FIT-based screening and a desire to detect lesions as early as possible. Shorter intervals were therefore readily accepted. Extended intervals raised concerns about missed lesions, but were largely accepted as individuals felt reassured by their low-risk classification. Moreover, nationwide implemented changes seemed to be readily accepted, with individuals trusting that these changes would only be implemented after scientific evaluation of their consequences.

The strong preference for an intensified screening approach over a less intensive screening approach aligns with previous research. Multiple studies that assessed the acceptance of hypothetical screening strategies concluded that extended intervals may be less acceptable than shorter intervals (Dunlop et al., 2021; Kvernrød et al., 2024; Kelley Jones et al., 2024; Loft et al., 2024; Ghanouni et al., 2020; Taylor et al., 2024; Meisel et al., 2015). Studies on risk-stratified breast cancer screening, for example, reported that only 42–59 % would accept less frequent screening when considered being at low-risk, while 85–89 % would accept intensified screening when considered being at high-risk (Loft et al., 2024; Ghanouni et al., 2020; Meisel et al., 2015). This preference for more intensive screening reflects participants' positive perception of cancer screening. In line with previous research, individuals generally perceived screening as highly beneficial, placing significant value on the sense of security and safety it provides, while showing less concern about potential harms and the burden of screening. (Dunlop et al., 2021; Laza-Vásquez et al., 2022; Dennison et al., 2023; Pluymen et al., 2023) Reducing screening for those at low risk to minimize these harms and burdens does not seem to align with the population's perception of cancer screening. In contrast, risk stratification may be better understood from the perspective of dividing resources between individuals with high and low risks, as offering less screening to individuals at lower risk to allocate more resources to those at higher risk was viewed as both reasonable and fair, as previously reported (Dunlop et al., 2021; Tan et al., 2025; Taylor et al., 2023).

Despite a general preference for shorter intervals, our findings demonstrate acceptance among participants assigned both shorter and extended invitation intervals. Perceived acceptance assessed in hypothetical settings may thus underestimate acceptance in a real-world setting. The feeling of reassurance individuals felt from being classified as low risk, also expressed by women classified as low- or medium-risk in breast cancer screening (McWilliams et al., 2023), was not reported by studies conducted in hypothetical settings. Knowing one's low-risk status appears to ease concerns about missed lesions with extended intervals and thereby facilitate the acceptance of less intensive screening. Moreover, concerns about missed lesions were partially confined to the research setting and are thus less likely to harm the acceptability of risk-stratification in a nationwide CRC screening program.

Although individuals generally understood the concept of risk-stratified screening, our results indicate that individuals' understanding of CRC risk is suboptimal. Individuals were generally unaware that CRC risk differs among those with a negative FIT, as the screening organization's communication of negative FIT results does not differentiate between individuals with no or minimal blood in their stool. Moreover, some individuals believed that a negative FIT meant they were not at risk for CRC. The dissatisfaction and confusion individuals assigned a one-year interval reported after being informed they were at higher risk highlights a strong desire for transparent and clear communication about FIT-results. If risk-stratification would be implemented nationwide, reasons for differentiating between individuals with a negative FIT, and therefore a different reporting of negative FIT-results, should be clearly explained to prevent confusion among the public. Another misconception identified is that part of the study population overestimated their ability to recognize symptoms caused by polyps or early-stage CRC, seeming unaware that these conditions can develop without causing symptoms or noticeable blood. While our study population was already participating in screening, this perceived control may cause individuals to underestimate the importance of participating in screening. Addressing this misconception in information materials may therefore increase individuals' understanding of the importance of participating in CRC screening. However, effectively informing the public remains challenging. Engagement with information materials is currently suboptimal, highlighted by the fact that individuals repeatedly suggested improving information materials by adding details already provided in current information leaflet of the screening program (e.g., red-flag symptoms to watch for between screening sessions). This emphasizes that future efforts should prioritize effective information delivery, balancing the provision of important content with keeping it manageable and understandable. Prior research already identified layered information provision as the preferred way to present information on risk-stratified screening, conveying key information to the public while offering additional details for those interested (Toes-Zoutendijk et al., 2023).

Our results show that individuals are less likely to question risk-stratified screening when implemented in the nationwide program compared to a research trial, placing trust in the provider of the screening program. In order to maintain this trust, the nationwide program should considerately communicate changes to the screening program, especially in regards to negative FIT-results. However, although high acceptance is highly favorable, prior research suggests that informed decision-making is hampered by participants tendency to view screening positively (Douma et al., 2019). Individuals who have already decided to participate in screening may heuristically accept changes to the program, such as risk-stratified intervals, without consciously weighing potential harms and benefits. Whether this also applies to risk-stratified screening remains uncertain until confirmed using a validated informed decision-making tool (Timmermans et al., 2024).

A key strength of our study is its evaluation of the acceptance of tailored invitation intervals within an ongoing risk-stratified screening program rather than a hypothetical setting. This approach minimizes hypothetical bias and provides a more accurate reflection of public acceptance in a real-world setting. Another strength is the inclusion of individuals who dropped out of the RCT. Interviewing these individuals offered unique insights in individuals' motives for not accepting less intensive screening, offering a more critical perspective. By including individuals assigned to both one- and three-year invitation intervals, we were able to provide a comprehensive overview of acceptance among those offered less and more frequent screening, highlighting differences between these groups. However, our research is not without limitations. Recruitment of study participants is prone to selection bias, as individuals who consent to participate in risk-stratified screening may possess an inherent tendency to view risk-stratified screening in a more favorable light than the general population. Moreover, the exclusion of non-Dutch-speaking participants and those without access to a mobile device may have introduced additional selection bias. Future studies should validate acceptance across the entire screening population. Furthermore, experiences and perceptions towards risk-stratification and cancer screening in general may differ in populations with different demographic, cultural, or healthcare contexts. Examining differences in acceptance by demographic factors such as age, sex, or socioeconomic status may offer more nuanced insights into the perspectives of specific subgroups. Additionally, individuals' responses may have been influenced by social desirability bias, though we believe this was minimized by efforts to create a conducive atmosphere (e.g. explicitly emphasizing we were interested in everyone's genuine experiences). Moreover, our transcripts and results were not returned to participants to validate that our findings accurately reflect their perspectives and experiences. However, we believe the impact of this limitation was minimized, as all researchers involved in data collection reviewed the findings to ensure they accurately represented the conversations. Finally, the perspective of individuals assigned to three-year intervals may change as they pass the two-year mark after their last screening, at which point they would typically be invited again. Therefore, adherence to risk-stratified screening should be evaluated once the RCT has concluded.

Transitioning from uniform to risk-stratified screening may raise concerns about individuals seeking screening outside of the organized program, particularly among those assigned extended intervals. In the Netherlands, this risk is limited as opportunistic screening is not facilitated. However, implications may be larger in countries with greater access to opportunistic screening. Without careful implementation, risk-stratified intervals could inadvertently lead to an overall increase in opportunistic screening rather than a more efficient use of resources, potentially tipping the balance between the benefits and harms of screening. Clear communication and involvement of key stakeholders may help mitigate these risks.

In conclusion, our findings demonstrate the high public acceptance of tailored invitation intervals in a population-based CRC screening program. This acceptance appears to be influenced by participants' trust in the screening provider, emphasizing the importance of maintaining this trust. Considerate communication about a changed way of reporting FIT-results, differentiating between individuals with a negative FIT-result, may facilitate the transition from uniform to risk-stratified invitation intervals.

## Disclosures funding

This study was funded by the Dutch Digestive Health Fund (MDL fonds) (WO 1944), as well as VIDI grant no. 09150171910047 from the Dutch Organization for Scientific Research (NWO). Both funding bodies had no role in the study design, data collection and analysis, decision to publish, or preparation of the manuscript.

## Data transparency statement

The data used for this study are confidential and cannot be shared.

## CRediT authorship contribution statement

**Brenda J. van Stigt:** Methodology, Formal analysis, Writing - Original Draft, Visualization. **Elyse E.C. Rijnders:** Formal analysis, Writing - Original Draft. **Lucie de Jonge:** Investigation, Writing - Review & Editing, Project administration. **Iris Lansdorp-Vogelaar:** Conceptualization, Funding acquisition, Methodology, Writing - Review & Editing. **Esther Toes-Zoutendijk:** Conceptualization, Funding acquisition, Methodology, Investigation, Writing - Review & Editing, Supervision. **Hilliene J. van de Schootbrugge-Vandermeer:** Investigation, Writing – review & editing. **Manon C.W. Spaander:** Conceptualization, Funding acquisition, Writing – review & editing. **Anneke J. van Vuuren:** Conceptualization, Writing – review & editing. **Evelien Dekker:** Conceptualization, Funding acquisition, Writing – review & editing. **Folkert J. van Kemenade:** Conceptualization, Writing – review & editing. **Iris D. Nagtegaal:** Conceptualization, Writing – review & editing. **Monique E. van Leerdam:** Conceptualization, Funding acquisition, Writing – review & editing.

## Declaration of competing interest

The following authors who are part of the Dutch colorectal cancer screening working group declare the following financial interests/personal relationships which may be considered as potential competing interests: Manon C.W. Spaander: Received research support from Sentinel, Sysmex, Medtronic and Norgine. Evelien Dekker: Honoraria for consultancy from Olympus, Fujifilm, Ambu, InterVenn, Norgine, Exact Sciences and ProMedCS. Speakers' fees from Olympus, Norgine, IPSEN/Mayoly, FujiFilm, Steris and Pentax. Endoscopic equipment on loan of FujiFilm. All other authors declare not conflicts of interest.

## Data Availability

The data that has been used is confidential.

## Appendix A Supplementary data

Supplementary material Image 1
