# Supplementary material for: Risk stratification in fecal immunochemical test-based colorectal cancer screening: Public acceptance and experiences with tailored invitation intervals
Source: Prev Med Rep. 2025 Nov 12;61:103310. doi: 10.1016/j.pmedr.2025.103310 (PMC12752790; doi:10.1016/j.pmedr.2025.103310)
Supplement: Supplementary file 1 — Supplementary material [file mmc1.docx]

**Supplementary Materials**

**Table of contents**

Supplementary figure

Supplementary file A: Provided information on risk-stratified invitation intervals

Supplementary file B: Informed consent form

Supplementary file C: Interview guide focus groups

Supplementary file D: Interview guide individual interviews

**Supplementary figures**


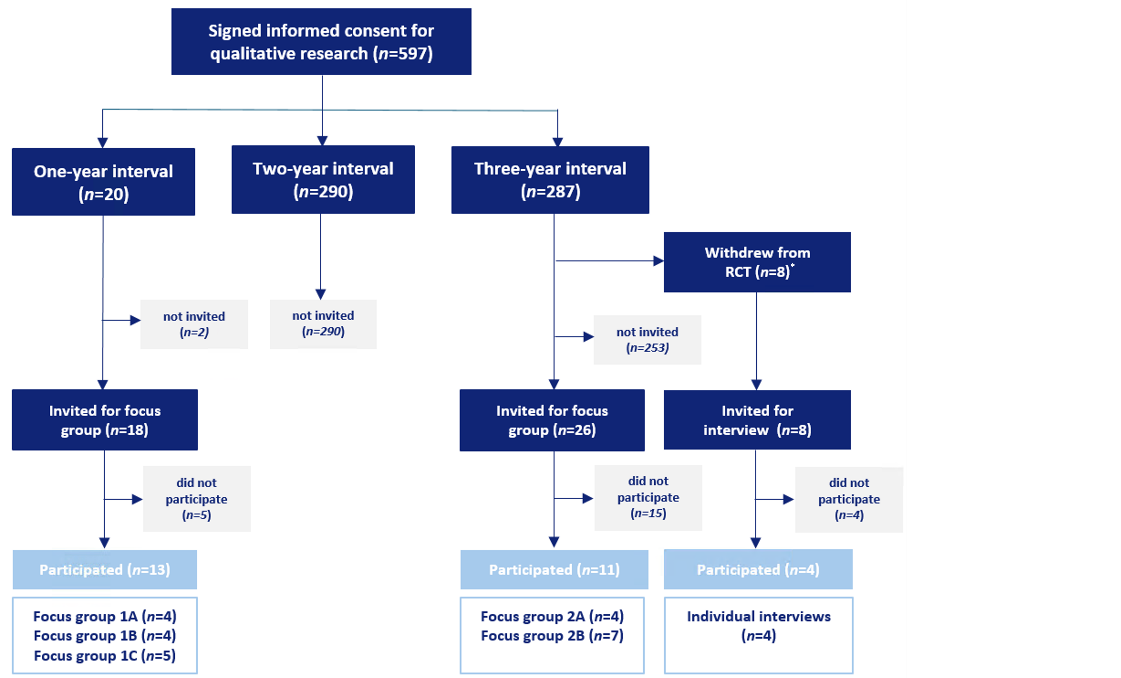


**Supplementary Figure 1:** Flowchart of the study population

*Number of individuals that withdrew from participation in the RCT before recruitment for the qualitative study.

*Abbreviation: RCT = Randomized Controlled Trial*

**Supplementary file A: Provided information on risk-stratified invitation intervals**

Information about personalized colorectal cancer screening, as included in the invitation letter of the PERFECT-FIT trial (translated to English), is shown below.

**Aim of the study**

Colorectal cancer screening has both advantages and disadvantages. The aim of this scientific study is to improve the balance between the pros and cons of colorectal cancer screening. We are conducting the research with people who received a favorable result from the previous stool test, meaning no follow-up examination was necessary. People like you. Until now, individuals with a favorable result were invited again two years later. They have a very low chance of colorectal cancer, but this risk can vary between individuals. Some people with a favorable result have a higher chance of developing colorectal cancer than others.

*Risk of colorectal cancer*

The risk of colorectal cancer depends on the amount of blood found in the stool. In this scientific study, we categorize this risk into three groups. Some people with a favorable result have no blood in their stool. Others have a small amount or a little blood. For those with a small amount of blood in the stool, the risk of colorectal cancer is slightly higher. Therefore, we would like to invite them earlier than after two years. This allows us to monitor them closely and treat them in time if necessary. We invite people after one, two, or three years. This means that you may be invited earlier or later. For some people, nothing will change.

*Disadvantages of colorectal cancer screening*

There are also disadvantages to colorectal cancer screening. We invite everyone after two years, including those who have no blood in their stool. Their risk of colorectal cancer is very low—lower than those with a small or very small amount of blood in their stool. Therefore, we want to invite them later in the scientific study, after three years. Some people find it stressful to wait for the test results, so reducing the frequency of invitations could be more comfortable for them.

*Personal risk of colorectal cancer*

Within this scientific study, we will no longer send a standard invitation after two years. Instead, you may receive an invitation after one, two, or three years, depending on your personal test results. You will receive an invitation that corresponds to your personal risk of colorectal cancer.

**Background of the study**

*How does colorectal cancer screening work?*

In colorectal cancer screening, you take a stool test. The screening organization checks whether there is blood in your stool. Every two years, you receive an invitation for a new test. The test result can be either favorable or unfavorable:

**Unfavorable result**: The laboratory detects blood in your stool. Follow-up research is necessary.

**Favorable result**: The laboratory detects no or very little blood in your stool. Follow-up research is not necessary. You will be invited again for another test in two years.

*How does this scientific study work?*

In the scientific study, we approach things differently. We are conducting the research with people who received a favorable result from the previous stool test. People like you. We invite participants after one, two, or three years, meaning you could be invited earlier or later. However, for some people, nothing will change.

**How does the study proceed?**

*How long does the study take?*

If you participate in the study, you will be offered a one-time "new" invitation interval. You will read more about this later. After participating in the study, you will still be invited for at least one more round of the "normal" colorectal cancer screening.

*What happens if I participate?*

If you participate in the study, you will receive a letter about six weeks later. This letter will specify when you will be invited for the next stool test. You will be placed either in the control group or the study group. This is determined by random selection. The researchers have no influence on this decision.

**Control group**: If you are in the control group, nothing changes for you. You will receive an invitation for a stool test again in two years, just like in the "regular" screening program.

**Study group**: If you are in the study group, the timing of your next invitation will differ. This depends on the results of your previous stool test.

- If your previous test showed no blood in your stool, you will be invited after three years.
- If your previous test showed a very small amount of blood in your stool, you will be invited after two years.
- If your previous test showed a small amount of blood in your stool, you will be invited after one year.

You will receive, as usual, a purple envelope with a stool test in the mail at your home address. You perform the test at home and send it to the laboratory.

**What are the advantages and disadvantages of participating in the study?**

Participating in the study may have both advantages and disadvantages. Below is a list of the pros and cons. Think carefully about them and discuss them with others.

*Advantages of participating in the study:*

1. We will no longer send a standard invitation after two years. Instead, you may be invited after one, two, or three years. The timing of your invitation depends on your personal results. You will receive an invitation that aligns with your personal risk of colorectal cancer, allowing us to assist you in the best possible way.
2. People without blood in their stool would typically be invited again after two years. However, since their risk of colorectal cancer is low, we will invite them later: after three years. For some, the reduced frequency of invitations is a relief.
3. An additional benefit is that fewer people will be unnecessarily invited for follow-up examinations. In these follow-up exams, it is often discovered that there is no colorectal cancer or precancerous condition, making the examination unnecessary. This is particularly inconvenient because there is a risk of complications during follow-up research.
4. People with a small amount of blood in their stool would typically be invited again after two years. Since their risk of colorectal cancer is higher than that of people with no or very little blood, we will invite this group earlier—after one year. This might allow us to detect or prevent colorectal cancer earlier.
5. People with very little blood in their stool have an average risk of colorectal cancer. For them, there is no change. They will continue to be invited after two years as usual.

*Disadvantages of participating in the study:*

1. It may happen that you receive an invitation to participate in the study after one year. This occurs if a small amount of blood is found in your stool, and we want to investigate it for safety reasons. We don't want to wait another two years. The likelihood is high that follow-up tests will show that no further investigation is needed. This is good news, but you might find it inconvenient to have undergone the test a year earlier for no reason.
2. If you participate in the scientific study, you may become worried. You will be informed of your personal risk of colorectal cancer, which may be slightly higher than that of others with a favorable test result. On the other hand, this means you will be invited earlier for the next test—after one year, rather than two years. If you do have colorectal cancer, we may detect it earlier.
3. It is also possible that you will not receive an invitation for three years, and in those three years, you might develop colorectal cancer or the early stages of it. The chance of this happening is very low (less than 16 out of 10,000 people), but it is still possible. You should always stay alert for symptoms. Common symptoms of colorectal cancer include unexplained and persistent changes in bowel movements, such as constipation or diarrhea, blood or mucus in the stool, fatigue due to anemia, abdominal cramps, and/or pain. These symptoms may indicate colorectal cancer, but they may also have another cause. If you experience any of these symptoms, contact your doctor.

**Supplementary file B: Informed consent form**

Please return this form within 3 weeks of receipt.

- I have read the information letter. I was also able to ask questions. My questions were answered satisfactorily. I had enough time to decide whether I want to participate.
- I understand that participation is voluntary. I also understand that I can decide to withdraw from the study at any time, without having to give a reason.
- I give the researchers permission to collect and use my data. The researchers will only use my data to answer the research question for this study.
- I understand that, for the purpose of monitoring the study, certain individuals may have access to my data. These individuals are listed in the information letter. I give these individuals permission to access my data for monitoring purposes.
- Please tick "yes" or "no" in the table below:

| **I give permission for my data to be stored and used for future research, as described in the information letter.** | Yes ☐ | No ☐ |
| --- | --- | --- |

- I agree to participate in this study.

Date of birth: …………………….

**Signature**: ……………………… **Date** : __ / __ / __

-----------------------------------------------------------------------------------------------------------------

**Only fill in the information below if you wish to participate in the group discussions (focus groups).**

| I would like to participate in the group discussions (focus groups) if I am selected for the study group. | Yes ☐ | No ☐ |
| --- | --- | --- |

**Only fill in the information below if you answered ‘yes’ to the question above.**

Name: …………………………………….

Phone number: ……………………….

E-mail adress:……………………………...

Adress:……………………………………..

-----------------------------------------------------------------------------------------------------------------

**Supplementary file C: Interview guide focus groups**

**Part 1: Decision to participate in scientific research**

1. What have been your reasons for choosing to participate in the scientific research?
2. Can you explain what the decision-making process looked like for you?
3. Did you discuss your participation with anyone?
4. What reasons could refrain you from participating?
5. Could you explain in your own words what the purpose of the scientific research is?
6. What do you believe the main reason is for moving towards personalized screening?

*Probes: costs, benefits.*

**Part 2: Screening based on personal colorectal cancer risk**

1. What do the terms 'favorable test result' and 'cut-off' mean to you?
2. How does it affect you to know that despite a favorable result, there might have been a little blood in your stool?

*Below, earlier and later are used for those assigned one- and three-year intervals, respectively.*

1. How did you feel when you received the letter saying that, based on your risk, you will now be invited 1 year earlier/later than two years as in the 'regular' population screening for bowel cancer?

*Probes: anxiety, worry, fear, relief*

- 1. How do you feel about being invited earlier/later than other people? What emotions does this evoke in you?

1. Can you explain in your own words why you were invited earlier to take the stool test?
   1. What do you think the advantages of this might be?
   2. What do you think the disadvantages of this might be?
2. Have you discussed your participation in the scientific study with anyone close to you? And that you have been invited earlier/later than you are used to?
3. Did this result influence your decision to participate in the population screening for colorectal cancer again?
4. What other actions, if any, did you take as a result of the letter? For example, did you contact you general practitioner? Or with anyone else?

**Part 3: Risk communication in colorectal cancer screening**

1. Can you remember how the result of the test was communicated to you?
2. Would you have needed more or less information about a favorable result?
3. How did you find the provision of information around the scientific study?
   1. What did you think was good?
   2. What would you have liked to know before your participation?
   3. Which information was unnecessary in your opinion?
4. How would you like it if the amount of blood and time to next screening were not communicated, so that you would only know at the time of the next invitation whether you will be invited after 1, 2 or 3 years?
5. Do you think this information could influence people's views on the outcome of population screening, and if so, how?
6. With the knowledge you have today, would you have preferred to stick with the original population screening without personalized screening anyway?
7. What changes, if any, would you like to make to make personalized screening even better?

**Part 4: Personalised screening in general**

1. What do you think about colon cancer screening based on personal risk as in this scientific study?
   1. Is it fair?
2. Would you advise family/friends to participate in a population screening study in which invitations are based on personal risk?
   1. Do you think it is well understood by everyone?
3. Does anyone have anything to add that has not been addressed?

**Supplementary file D: Interview guide individual interviews**

**Part 1: Decision to participate in scientific research**

1. What have been your reasons for initially choosing to participate in the scientific research?
2. Could you explain in your own words what the purpose of the scientific research is?
3. What do you believe the main reason is for moving towards personalized screening?

*Probes: costs, benefits.*

**Part 2: Screening based on personal colorectal cancer risk**

1. How did you feel when you received the letter saying that, based on your risk, you will now be invited 1 year later than two years as in the 'regular' population screening for bowel cancer?

*Probes: anxiety, worry, fear, relief*

- 1. How do you feel about being invited later than other people? What emotions does this evoke in you?

1. What other actions, if any, did you take as a result of the letter? For example, did you contact you general practitioner? Or with anyone else?
2. What was your reason for opting out after receiving the letter that you will be invited after three years?

**Part 3: Risk communication in colorectal cancer screening**

1. Would you have needed more or less information about a favorable result?
2. How did you find the provision of information around the scientific study?
   1. What did you think was good?
   2. What would you have liked to know before your participation?
   3. Which information was unnecessary in your opinion?
3. How would you like it if the amount of blood and time to next screening were not communicated, so that you would only know at the time of the next invitation whether you will be invited after 1, 2 or 3 years?
4. What changes, if any, would you like to make to make personalized screening even better?

**Part 4: Personalized screening in general**

1. What do you think about colon cancer screening based on personal risk as in this scientific study?
   1. Is it fair?
2. Would you advise family/friends to participate in a population screening study in which invitations are based on personal risk?
   1. Do you think it is well understood by everyone?

Do you have anything to add that has not been addressed?
